# Tracing fineware production in the Neo-Assyrian empire: Neutron activation analysis of common and Palace Ware in the upper Tigris River Valley, Turkey

**DOI:** 10.1371/journal.pone.0315378

**Published:** 2025-01-07

**Authors:** Britt E. Hartenberger, James A. Davenport, Timothy Matney

**Affiliations:** 1 Institute for Intercultural and Anthropological Studies, Western Michigan University, Kalamazoo, Michigan, United States of America; 2 Archaeometry Laboratory, University of Missouri Research Reactor, University of Missouri, Columbia, Missouri, United States of America; 3 Department of Anthropology, University of Akron, Akron, Ohio, United States of America; Israel Antiquities Authority, ISRAEL

## Abstract

In the Iron Age, the Neo-Assyrian empire (c. 900–600 BC) conquered territory across southwest Asia and established regional capitals along its borders to secure its gains. Governors at these centers oversaw resource extraction and craft production for shipment to the imperial heartland in modern-day northern Iraq. Metals and textiles were the crafts most carefully managed by the administration. We know less about centralized control over ceramic production but hypothesize that fineware production and distribution would have been of interest to imperial administrators. A fineware type known as Palace Ware has been found throughout the empire and is considered an indicator of elite Assyrian dining traditions. Excavations at one regional capital, Ziyaret Tepe (ancient Tušhan) produced pottery of various skill levels used by residents. In this study neutron activation analysis (NAA) was used to characterize and compare the fabrics used to make Palace Ware vessels with more common wares to see if the former vessels were imported from the imperial heartland. Palace Ware is macroscopically distinct, but this does not always indicate an import. Chemical composition of the samples fell into four main groups, and both Palace and common ware were found to have similar compositions. Comparison of these data with those from contemporary sites showed that the two main Ziyaret groups matched the chemical composition of pottery from the Assyrian capitals of Nimrud and Nineveh. Our conclusions show that there is considerable homogeneity in the clays of the upper Tigris river valley in Turkey and the lower Tigris in northern Iraq. Given this similarity, it is possible that Palace Ware at Tušhan was produced locally, imported, or both. If it was manufactured locally, as has been shown at the urban center of Tell Sheikh Hamad, potters in the imperial peripheries may have produced fineware pottery independent of direct imperial control.

## Introduction

Pottery is often used in archaeology as an indicator of craft production or trade in raw materials, as well as for its basic chronological use in charting stylistic change over time. In the context of Iron Age greater Mesopotamia (12^th^ to 7^th^ centuries BC), we know from textual records and finds of raw materials like metal ores outside their source area that significant trade took place. Craft production can be more difficult to trace because workshops are rare finds on large sites as modern excavations often sample only a small proportion of the site area. Contemporary cuneiform texts, while primarily economic, are more concerned with government control of valuable items, usually metals, textiles, or basic foodstuffs (especially grain and flocks). When ceramics are mentioned at all, it is often to discuss the edible contents of ceramic jars and not jars and bowls themselves. Assyrian military expansion and conquest often involved a re-organization of political control and economic production, the latter including standardization of specialist craft production [1]. It is unknown to what extent pottery production was controlled by the imperial administration, especially in the Neo-Assyrian period (ca. 934–611 BC) [2].

In past decades, archaeologists hypothesized where pottery production took place based on macroscopic clues such as style, form, and decoration. These features can be misleading especially in the case of local imitations of foreign-made wares. Ethnographic research has shown that potters usually use the clay source closest to them for production, so clay composition would be another way to distinguish production locales [3]. Provenience studies in pottery are possible because clay composition differs more significantly between geological regions than within a single geological source. Low-power microscopic analysis of the petrography of the clay body is one way to characterize the geologic differences in clays. Modern chemical provenience studies such as Neutron Activation Analysis (NAA) can characterize pottery accurately by detecting its elemental composition down to the parts per million. During its construction, other materials (‘temper’) are usually added to the clay body of a vessel to make the clay easier to shape and improve its firing characteristics. A study of its chemical composition, both major and trace elements, will detect characteristics of its clay as well as the additional temper added by the potter.

### Ziyaret Tepe

**Fig 1:**
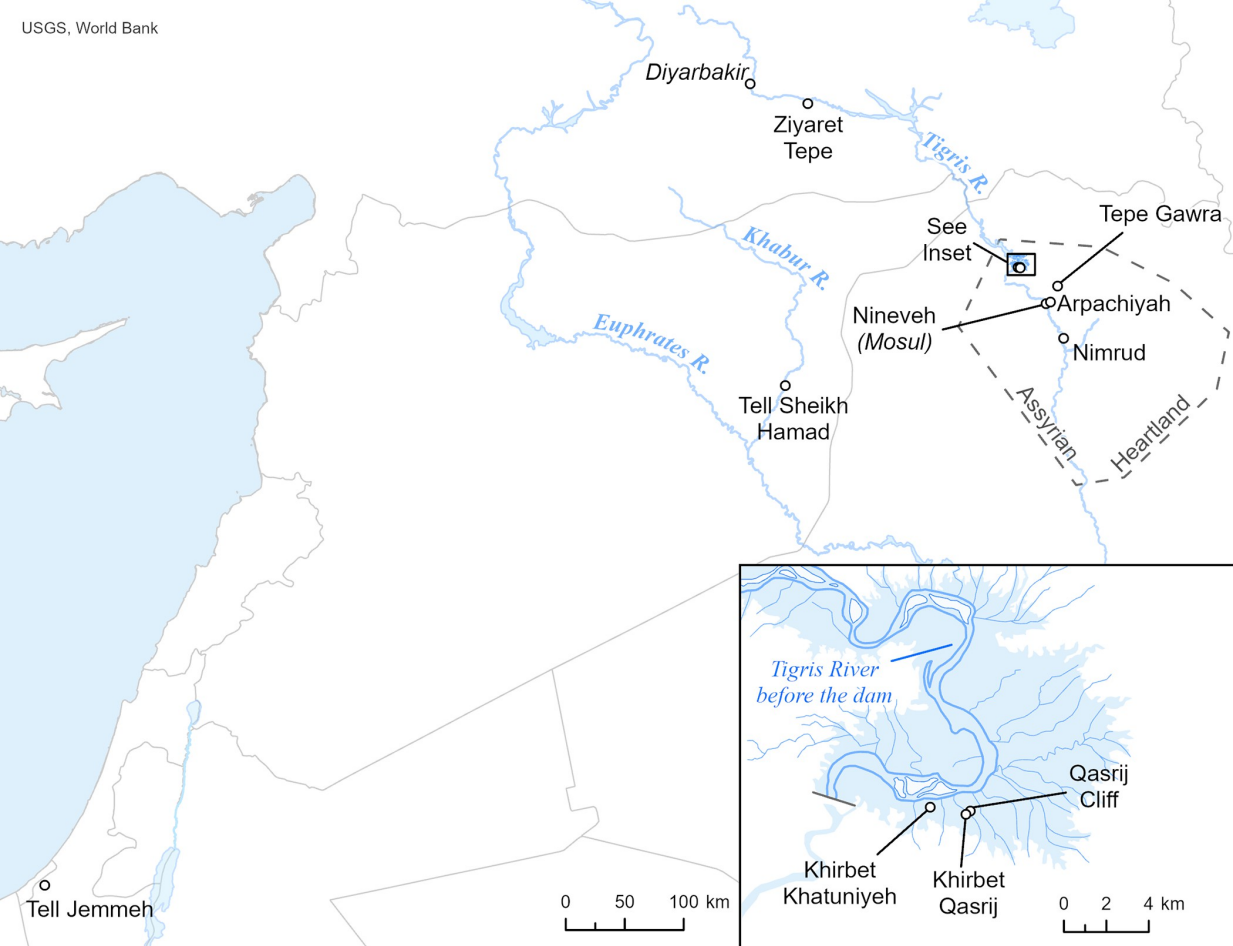
Map showing sites mentioned in the text. (Credit: World Bank; U.S. Geological Survey, Department of the Interior/USGS, U.S. Geological Survey Global GIS Database, image by Paul Hearn, T.M. Hare, P. Schruben, D. Sherrill, C. LaMar, and P. Tsushima).

From 1997 to 2014, the Ziyaret Tepe Archaeological Project investigated an ancient mounded site in southeastern Turkey [4]. The site has a series of occupations extending back to the Early Bronze (c. 3000 BC), Middle Bronze, and Late Bronze Ages, reaching a maximum extent as a provincial capital of the Neo-Assyrian empire in the Iron Age (900 to 600 BC) when the urban site (32 ha) was called Tušhan. Tušhan was located approximately 270 km northwest (although 425 km by river transport) from the Assyrian homeland in northern Iraq (Fig 1). Texts from King Ashurnasirpal II (ruled 883–859 BC) describe three regional capitals being established as the northern frontier along the Tigris River: Tušhan, Sinabu, and Tidu. The Assyrians installed a governor at Tušhan and built a palace for him, garrisoned troops there, and built an encircling city wall. Cuneiform texts found in its palace and administrative buildings detail the military, economic activities such as food and textile production, and governance of the site [5, 6]. Smaller sites nearby specialized in grain production and work groups stationed at Tušhan supplied the empire with materials from this region, especially timbers from the nearby mountains that were floated down the Tigris [7], and likely funneling metal ores from mountain sources to the Assyrian heartland. Later occupational levels at Ziyaret Tepe dated to the Late Iron Age, Medieval, and Ottoman periods.

**Fig 2:**
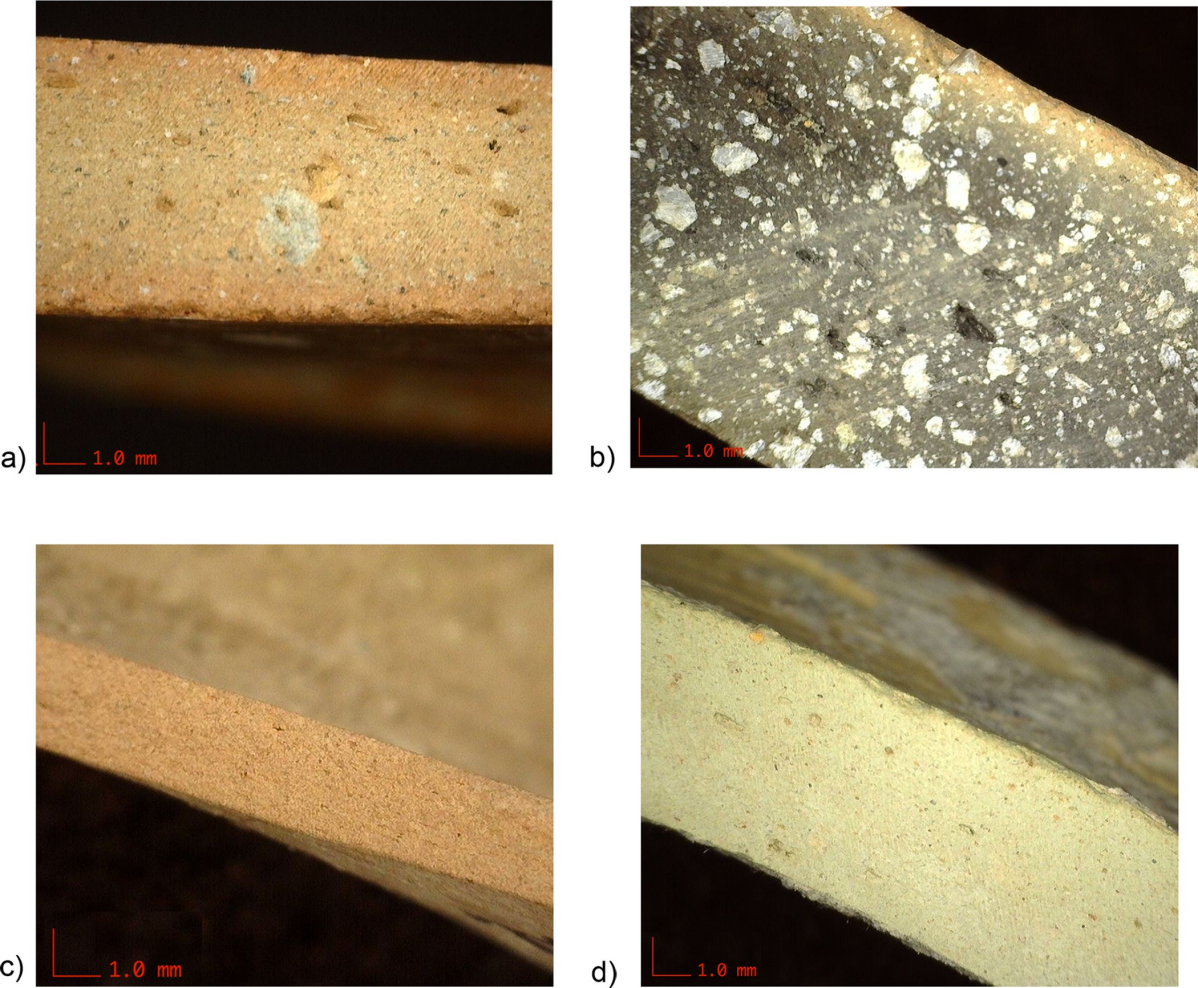
Illustration of a) Plain Simple Ware (LA01), b) one of the cooking wares (LA03), c) Palace Ware (LA05) and d) Near Palace Ware (LA06), at 20x magnification. The sherds are a) ZT 8186/1, b) ZT 11707/2, c) ZT 48352/1, and d) ZT 5738/3 (photographs by BH).

Several different types of pottery wares are found across the excavated buildings and burials at Neo-Assyrian period Tušhan, differing in skill level, appearance, and function. The most common ware is called Plain Simple Ware (LA01) in our recording system. It is medium in coarseness with multiple different kinds of temper and was fired to a light reddish-brown to buff color. It is found in a variety of jar and bowl shapes. Other wares include two cooking wares (LA03 and LA04) used to create large globular pots, as well as finewares such as Palace Ware (LA05), a Near Palace Ware (LA06), and a rarer Neo-Assyrian glazed ware (LA10). Fig 2 shows examples of these wares.

### Palace Ware

Palace Ware is thin, fine-grained, and made of well-levigated clay; its manufacture is a highly skilled technique. Palace Ware vessels come in a set of standard shapes like a dining set [8, 9]. This sophisticated type was found in low quantities in most domestic Neo-Assyrian contexts at Tušhan and visually mirrors pottery used in Assyrian capitals. It is absent in the preceding Early Iron Age contexts when a small part of the high mound at Ziyaret Tepe was used by indigenous groups between 1050 BC and the Neo-Assyrian conquest in the early 9^th^ century BC. Palace Ware was first defined in the 1950s after numerous examples were found at the Assyrian capitals [10, 11] and it is only found in a narrow time period (late 9th century BC through the end of the 7th century BC). Wherever Palace Ware appears, whether it is in Syria, Iraq, Israel, or Turkey, it is regarded as an indicator of Neo-Assyrian influence [2, 12]. Three basic shapes of Palace Ware have been defined using measurements of vessels from the capitals of the Neo-Assyrian empire (Nineveh and Nimrud), and all are drinking-related: bowls, cups, and small jars [9]. We have found examples of all three of these forms at Tušhan [13]. Their overall frequency is a small percentage of the pottery assemblage (1–7% depending on the context), but even in small quantities it is significant as an indicator of a “foreign” dining tradition in a provincial context.

Our hypothesis was that, given the fragility of Palace Ware for travel, we expected to find that potters were producing both Palace Ware and common wares using local Upper Tigridian clay sources in workshops located at, or in the immediate vicinity of, Ziyaret Tepe. This NAA study cannot directly address the possibility that the potters themselves were resources and could have been moved into the region from the imperial homeland, either to produce or to train other potters in elite, Palace Ware production techniques.

## Materials and methods

At other sites, it has proven difficult to distinguish Palace Ware that is imported from local imitations. Some local potters copied the type if they were skilled enough to do so because it was a luxury good, presumably of greater value. Imitation pieces have been found in Palestine, Transjordan, and Syria [2, 14, 15]. In attempting to source Palace Ware from sites on the edges of the empire, techniques such as ceramic petrography have been used but, by itself, this method was not always successful in distinguishing between clays at sites, and chemical methods have proven more useful. For example, Hunt was able to distinguish clays along the Euphrates River from those along the Tigris using NAA due to their slightly different clay minerals [2].

One significant issue addressed in this study is the relative homogeneity of geological formations along the Tigris River, discussed below. As a result, in some cases it has not been possible to distinguish clay fabrics between nearby sites on the Tigris, e.g., when comparing pottery from Arpachiyeh and Tepe Gawra to that found at Khirbet Qasrij and Qasrij Cliff 25 km to the south, or nearby Khirbet Khatuniyeh [16, 17]. Since Tušhan is 425 km upstream from this immediate area around Nineveh, we hoped to find a distinctive clay chemical signature for common ware at the site that would contrast with the known signature for Palace Ware as already defined in the imperial heartland in the studies cited above.

### NAA sampling

Several studies have used NAA to compare the chemical characteristics of pottery from Tell Sheikh Hamad (ancient Dur-Katlimmu), Tell Jemmeh (in Israel), and from two Neo-Assyrian capitals, Nimrud and Nineveh [2, 18]. Tell Sheikh Hamad was contemporary with Tušhan and also functioned as a regional Neo-Assyrian provincial capital. It is located about 230 km to the southwest of the Assyrian heartland on the lower Khabur River, a tributary of the Euphrates. Wares used at Tell Sheikh Hamad included a version of our Plain Simple Ware as well as a fine-grained ware “B1” which is their equivalent to Palace Ware [19]. Based on Hunt’s petrographic and chemical analysis of the pottery, she concluded that potters at Tell Sheikh Hamad made their own version of Palace Ware using local clays that looked very similar to examples made in the capital cities [2]. Hunt used geologic methods and chemical methods such as NAA to characterize Palace Ware at the capitals of Nimrud, Nineveh, and Aššur as her baseline for comparison [9, 18]. Her results are discussed below in relation to our own chemical analyses on the Ziyaret Tepe samples.

A total of 50 pieces were selected for analysis from the exported sherds taken from the Ziyaret Tepe excavations (see Table 1 below). All necessary permits were obtained from the Diyarbakır Museum and the Turkish Ministry of Culture and Tourism for exporting the potsherds for the described study, which complied with all relevant regulations. These included 40 samples of probable local pottery. We used a standard concept called the ‘criterion of abundance technique’ to characterize the local pottery samples from the site [20]. Simply stated, we can safely assume that Plain Simple Ware is local because it makes up the majority (81% in primary contexts) of Iron Age pottery at the site and there would be no need to trade or import ordinary pottery or cooking wares from elsewhere. Also as noted above, potters typically use clay found near their workshop location. Plain Simple Ware provides one indicator of local clay chemical signatures. Another source of information on the clays used are the discarded mistakes from pottery kilns at the site, overfired pieces called ‘wasters’.

**Table 1:** List of samples by ware, quantity, and period.

Ware type	Ware name	No. of samples	Period
LA01	Plain Simple	10	Neo-Assyrian
LA03 and LA04	Cooking	10	Neo-Assyrian
LA05	Palace	6	Neo-Assyrian
LA06	Near Palace	3	Neo-Assyrian
ER01	Plain Simple	4	Early Iron
ME03	Cooking	11	Medieval
MB03	Cooking	1	Middle Bronze
Waster	unknown, vitrified	3	Medieval
Waster	unknown, vitrified	1	no date
XX	Cilician ware?	1	Iron Age?

Some contexts from which our samples are drawn date from other time periods than the Iron Age but are assumed to have used the same local Upper Tigridian clays in their production as did the potters of the Neo-Assyrian period. These include: one Middle Bronze Age cooking ware sherd, four Early Iron Age Plain Simple Ware sherds, and eleven Medieval cooking ware sherds. The one piece of Cilician Ware, stylistically a clear foreign import found in a single primary context in the palace at Tušhan, was included. Painted pottery is rare in the Neo-Assyrian period and comparanda suggest that this painted piece may be an import from Cilicia, 500 km to the west of Ziyaret.

We did not collect modern clay samples from the region during the project. Our permit for the Ziyaret Tepe project was limited to on-site mapping and excavation and did not include survey of the surrounding area so we did not map nearby clay sources while in the field. The composition of the local clay, however, can be hypothesized from the geological context. Geologically, the upper Tigris River flows through Lower Miocene and Upper Miocene-Pliocene rock formations [21, 22]. The Lower Miocene limestone and sandstone formations contain abundant quartz, feldspar and silt and the Upper Miocene-Pliocene formations are conglomerates, clay, and silt [21]. At its upper elevations the Tigris also flows through pre-Neogene limestones composed mostly of calcium carbonate in the form of calcite and ophiolitic mélanges containing sedimentary and igneous rocks [21]. Given this geologic signature of the region, we expected NAA to show high amounts of calcium, silicon, and likely iron and sodium and/or potassium from the feldspars in the local clays.

Since pottery contains temper added by the potter, we also expected to find some elements deriving from the mineral or organic inclusions visible in cross-sections of the sherds. One common temper is grain chaff, and other types frequently seen in the clay macroscopically are white quartz grains, mica, and black, white, or red mineral inclusions. From a macroscopic perspective, it is not possible to identify these minerals, except that we may hypothesize feldspar, quartz, or crushed conglomerate from the nearby river deposits. Previous petrographic and chemical (X-ray fluorescence) study of common ware pottery from the Upper Tigris region has indicated it often contains quartz and muscovite inclusions and that the local clays are iron-rich [23].

### NAA methods

NAA was conducted by the Archaeometry Laboratory at the University of Missouri Research Reactor (MURR) using the standard methods and parameters at that laboratory. These methods are described in detail elsewhere [24–26]. To briefly summarize, a fragment of roughly 1 cm^2^ was removed from each sherd. Because NAA is a bulk analytical technique, all surfaces were removed by burring using a silicon-carbide grinding tool to account for any compositionally distinct surface treatments, like clay slips or pigments applied as decoration. This also accounts for any post-depositional contamination from taphonomic processes. After burring was completed, samples were rinsed in deionized water and allowed to dry. Samples were then homogenized into a fine powder through grinding with an agate mortar and pestle and placed in a drying oven to remove any remaining moisture in the samples for a minimum of 24 hours at 105°C. Once completely dry, aliquots were measured into two vials: 100 mg of powder was measured into a high-density polyethylene vial, and 200 mg of powder measured into a high-purity quartz vial and sealed under vacuum. Masses were recorded to the nearest 0.01 mg, and all values were within ± 2 mg of the target mass.

Two at a time, the aliquots in the polyethylene vials were loaded into a larger polyethylene container called a ‘rabbit’ and transported to the reactor via a pneumatic tube system for an irradiation of five seconds by a neutron flux of 8x10^13^ n cm^-2^ s^-1^. During this process, three samples of standards of certified reference material from NIST of SRM1633c Coal Fly Ash and SRM688 Basalt Rock, and an in-house quality control of New Ohio Red Clay were also irradiated under the same parameters. After being allowed to decay for 25 minutes, samples were counted for a period of 12 minutes by high-purity germanium detectors, yielding values in parts per million for nine elements: Al, Ba, Ca, Dy, K, Mn, Na, Ti, and V.

Aliquots in quartz vials were bundled into groups of 50 samples along with four samples of standard SRM1633c, and quality controls of SRM679 Brick Clay and New Ohio Red Clay. These bundles were irradiated for a period of 24 hours in a neutron flux of 6 x 10^13^ n cm^-2^ s^-1^. After an initial decay of seven days, these samples were washed and detected by high-purity germanium detectors for a period of 30 minutes each, yielding counts for As, La, Lu, Nd, Sm, U, and Yb. Samples were then allowed to decay for an additional two weeks before a second detection period of 2.5 hours, yielding counts for Ce, Co, Cr, Cs, Eu, Fe, Hf, Ni, Rb, Sb, Sc, Sr, Ta, Tb, Th, Zn, and Zr.

After all three periods of detection were complete, datasets were assembled and evaluated using a suite of multivariate statistical routines that are commonly applied to compositional data of archaeological ceramics and other materials [27–31]. This began with a calculation of a total variation matrix (TVM) [32–34], a table composed of log-transformed data where each element is expressed as a ratio of all other elements in the dataset. Total variation (*vt)* is the sum of all variances in the variation matrix divided by twice the number of elements in the matrix [34]. This value provides a metric to evaluate variability in a chemical dataset which is compatible with both variances and Euclidean distances. This value is significant to the evaluation of ceramic composition studies as it is an indicator of what is referred to as a monogenic or polygenic datasets. A high value indicates a polygenic dataset. For a study of ceramic composition, this translates to multiple compositional groups made from chemically discrete raw materials.

Groups were next identified using a combination of different statistical methods which are commonly used in the interpretation of compositional data of archaeological ceramics [20], including principal components analysis (PCA), hierarchical cluster analysis, and total variation matrix. PCA demonstrated that greater than 95% of the cumulative variance can be explained by the first eight principal components. Sherds were assigned into four distinct compositional groups, with one outlier. After group assignments were made, group membership was evaluated and refined through the calculation of Mahalanobis distances. After group assignments were made, group members were examined across different attributes, including ware and time period.

The Archaeometry Laboratory at MURR maintains a database of compositional data of archaeological objects and source materials, including over 300,000 archaeological ceramics. Additionally, the Archaeometry Laboratory curates data from other reactors, some of which are no longer operational and others that no longer use NAA on archaeological materials. NAA data from this research was compared to relevant datasets from these databases. To compare to data from Hunt and Sterba [18] analyzed at the Technische Universität Wien, it was necessary to calculate a new PCA, removing values from elements that were not detected in common between the two reactors (aluminum, calcium, dysprosium, manganese, titanium, vanadium, and tungsten).

## Results

The chemical composition of the 50 sherds was detected by element. The University of Missouri Research Reactor (MURR) then described these patterns with various statistical techniques. In the calculation of the total variation matrix, chromium (Cr) showed highest amount of variation while dysprosium (Dy) showed the least. The TVM of the samples has a total variation (*vt*) value of 4.405. Often the integer is equivalent to the amount of groups present in a single dataset, so a *vt* value of 4.405 suggests that this data is polygenic and is made up of at least four compositionally discrete groups.

**Fig 3:**
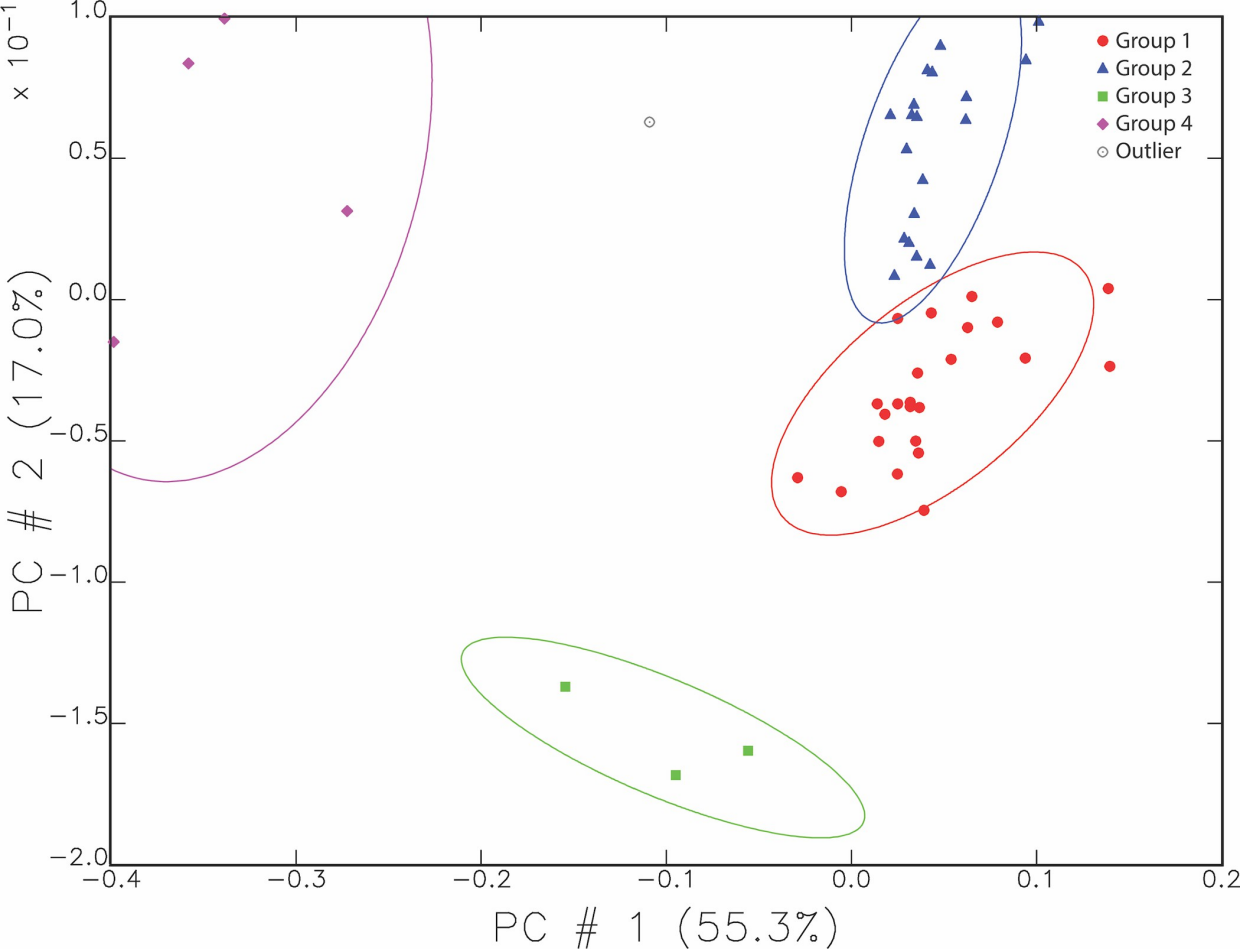
Main compositional groupings of the Ziyaret Tepe samples. The scatterplot shows the sample distribution using the first and second principal components representing 72.3% of the total variance. The ellipses are drawn at 90% confidence intervals.

The chemical compositions of the samples cluster statistically into four main groups and four outliers in the principal components analysis. Fig 3 shows this pattern using the first two principal components, and accounts for 72.3% of the variation in the data. Groups 1 and 2 contain the majority of the pottery and are therefore assumed to represent the chemical signature for local clays. They are somewhat distinct from each other though they vary more from Group 4 (purple) and Group 3 (green).

**Fig 4:**
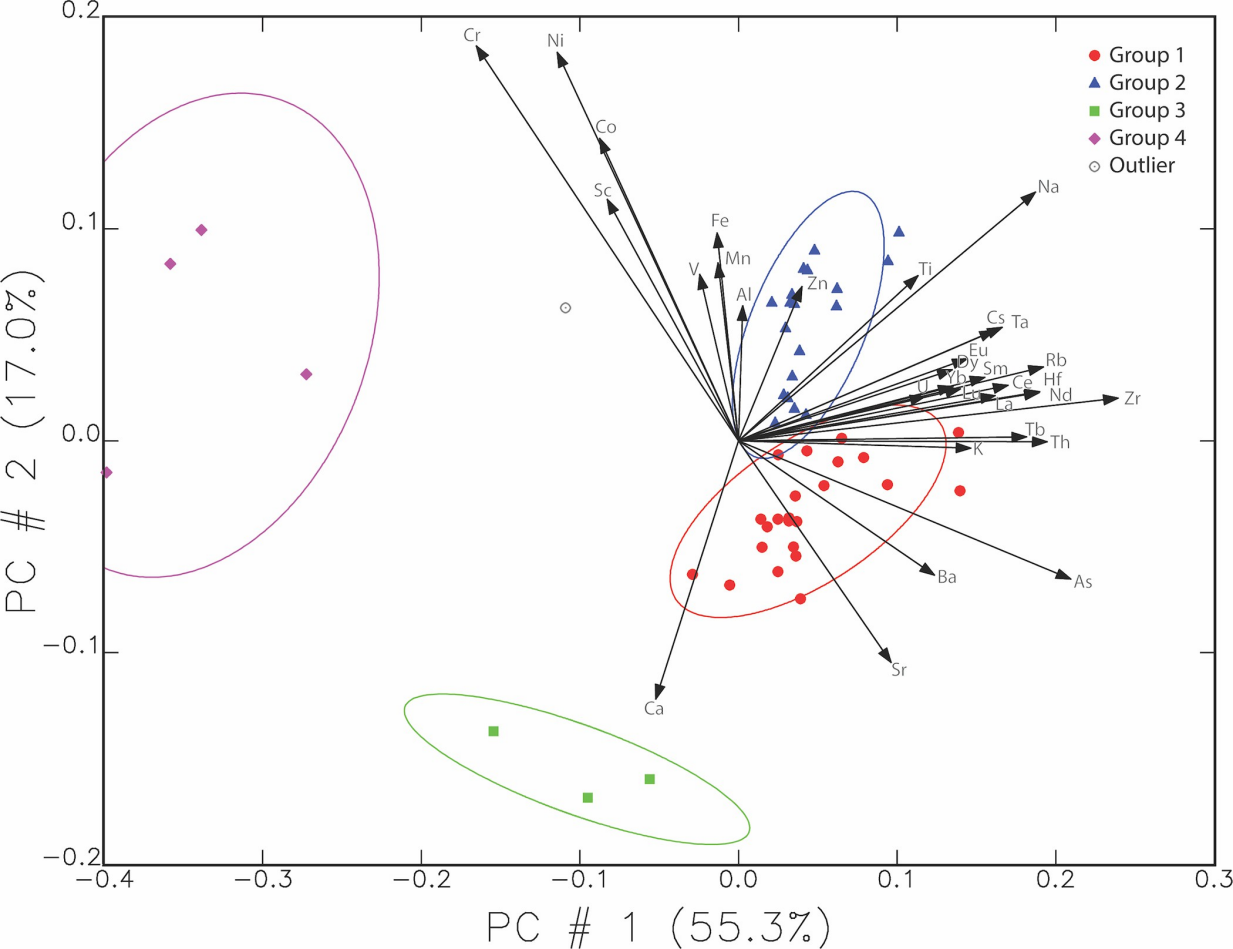
Biplot showing the distribution of samples using the first and second principal components with elemental vectors added. Ellipses are drawn at 90% confidence intervals.

Another view of this data can be seen in Fig 4 below, where the elemental vectors in the principal components analysis are included. Chromium, as the element contributing the most difference, has a noticeably long vector compared to most other elements, with nickel being the second longest.

**Fig 5:**
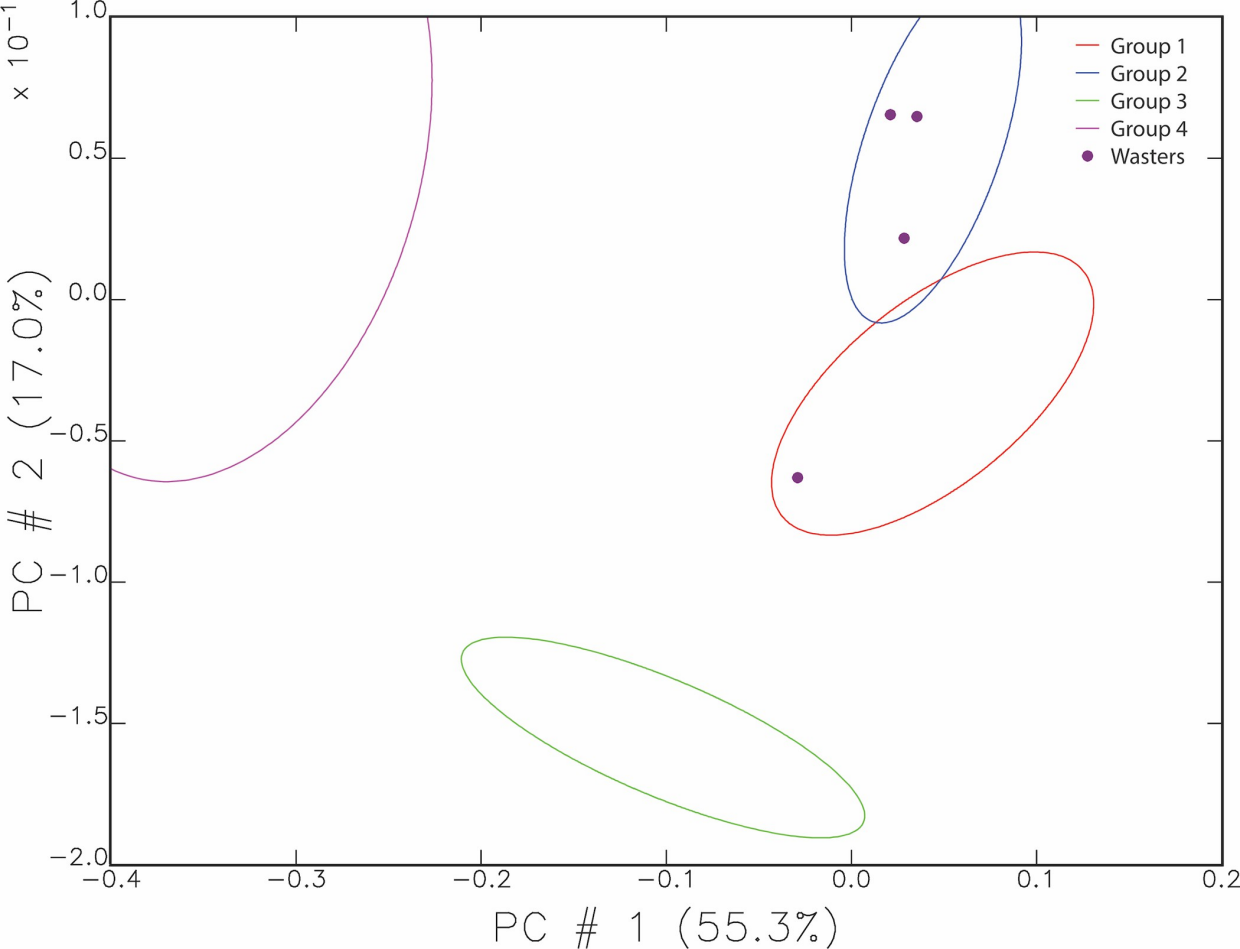
Graph of primary and secondary principal components, showing wasters plotted as purple dots.

Fig 5 graphs the compositional groups again, showing the four wasters as purple dots, showing that all wasters fall within either Group 1 or 2. Three wasters are from Medieval contexts and one from an undated context. The one waster seen in Group 1 is medieval in date.

**Fig 6:**
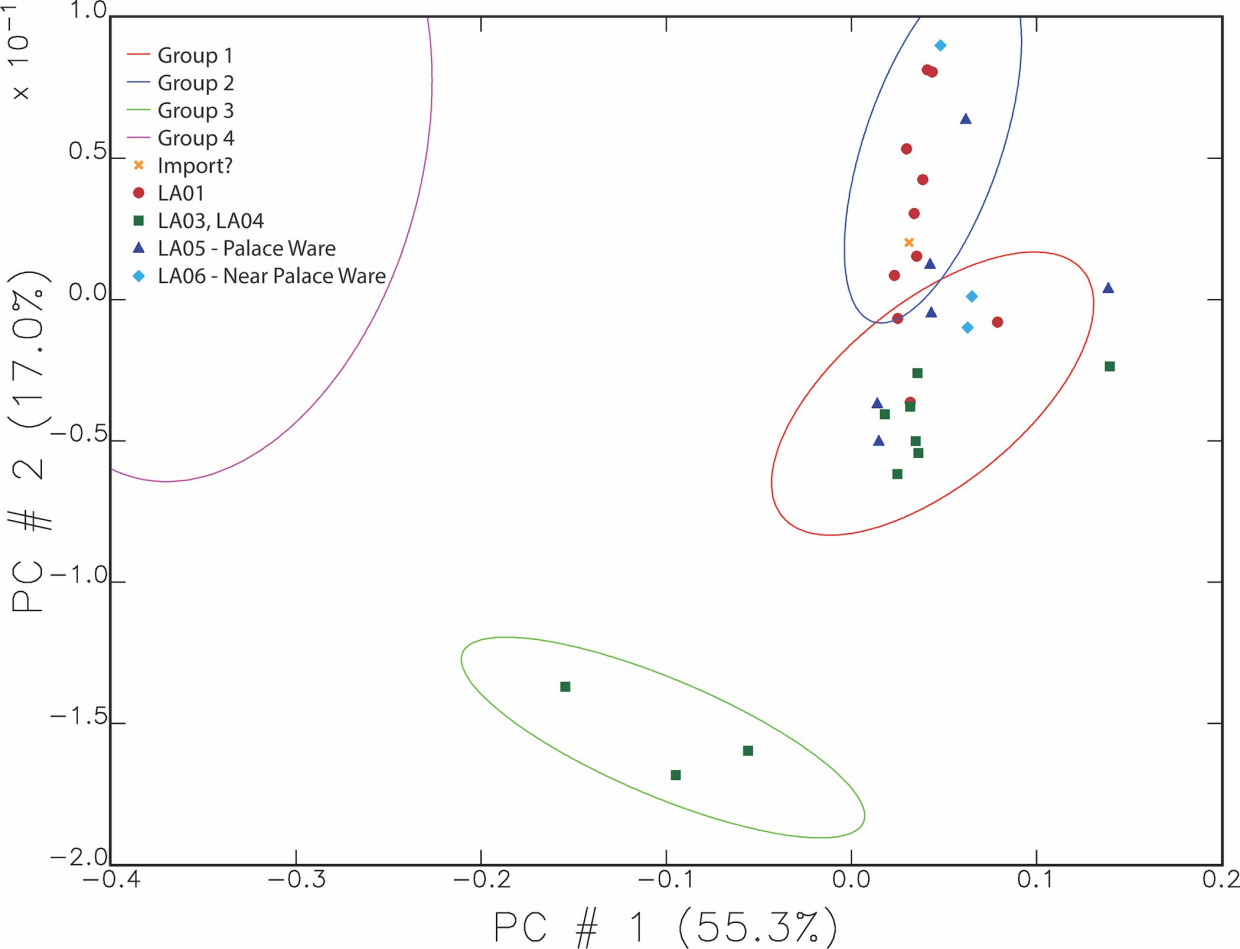
Graph of the primary and secondary principal components showing compositional groups as ovals with symbols for the Iron Are ware types. The ellipses indicate 90% confidence intervals.

Though the scatterplots above are useful for describing the local clay signature near Ziyaret, they do not take the time periods or wares of the samples into account. When mapped by our defined wares for the site, the Iron Age wares fall into Groups 1, 2, and 3 but not 4. Fig 6 shows the same composition groups as oval border lines and in this case the symbols indicate the Iron Age samples only. The possible import from Cilicia is coded as “Import?” in this plot.

Nearly all the Iron Age samples fit into or fall near Groups 1 and 2. One pattern visible in Fig 6 is that all the samples within Group 3 are cooking wares (LA03 and LA04), represented by green squares. Other samples of cooking wares are also present in or near Group 1. MURR determined that the key distinguishing element separating Group 3 from Groups 1 and 2 was calcium. Group 3 pots contained 18–21% Ca compared to Groups 1 and 2, where Ca levels were between 5 and 10%. When the additional Ca was corrected for and the principal components analysis run again, those three samples then fell within Group 1. Therefore, these three cooking pots (out of 10 Neo-Assyrian cooking ware samples) were made using the same clay as other pottery at the site, but with a higher level of calcium. The only macroscopic difference in cooking wares to suggest a higher calcium content was a common occurrence of medium white mineral inclusions in these wares, compared to a common prevalence of fine white mineral inclusions in Plain Simple Ware.

With Fig 6, we can see where the Palace Ware (LA05 and LA06) fits as compared to the local chemical signature indicated by Groups 1 and 2. Most of the Palace Ware pieces sampled match the chemical composition of the local pottery as represented by the Plain Simple and cooking wares from Ziyaret composing Groups 1 and 2. Compositionally, the fine wares cannot be distinguished from the chemical signature of the local pottery through the statistical analyses that were applied.

### Comparisons with other sites

**Fig 7:**
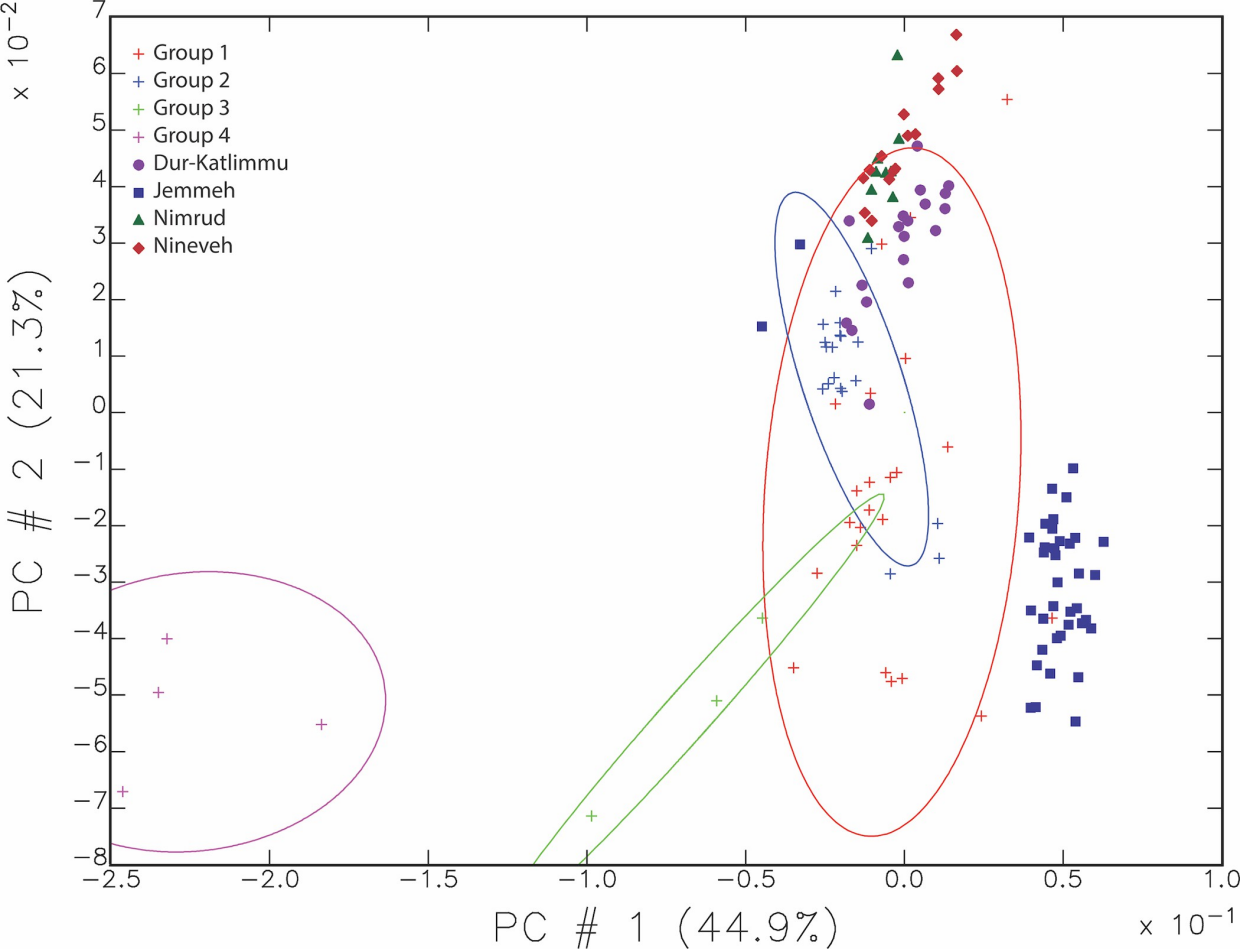
Scatterplot of principal components 1 and 2, representing 66.2% of the total variance in the data. Individual samples from Ziyaret are shown as plus signs while the samples from the other sites analyzed at the Vienna lab are other symbols.

The chemical composition of pieces from Ziyaret was compared with other regional capitals and the imperial capitals of Nineveh and Nimrud in the heartland of the empire using the data of Hunt and Sterba [18]. The other two regional capitals are relatively nearby, ancient Dur-Katlimmu (modern Tell Sheikh Hamad in Syria) and on the outskirts of the empire, Tell Jemmeh in modern Israel [18]. A PCA calculated with the 25 elements detected in common between the Missouri and Vienna reactors demonstrated that greater than 95% of the cumulative variance can be explained by the first nine principal components. Fig 7 shows the PCA when all these samples are combined, graphed by the resulting first and second principal components.

Much of these data overlap in Fig 7 except the Tell Jemmeh (blue squares) samples which are more distinct from the others. Ziyaret Group 1 as outlined in red contains a broad area in this scatterplot and overlaps with most of the samples from Dur-Katlimmu (purple dots). Nineveh and Nimrud are closer to each other and further away from Tell Jemmeh than Ziyaret Group 1. Given that Tell Jemmeh is in Israel, the chemical signature of its clay is quite different than the other sites. Dur-Katlimmu is on a tributary leading into the Euphrates and so may be expected to be significantly different from the others along the Tigris, but in fact falls within Group 1 from Ziyaret. Group 2 from Ziyaret is a small area in this plot and does not contain many samples from other sites and in particular does not contain any of the signatures of samples from Nineveh and Nimrud.

**Fig 8:**
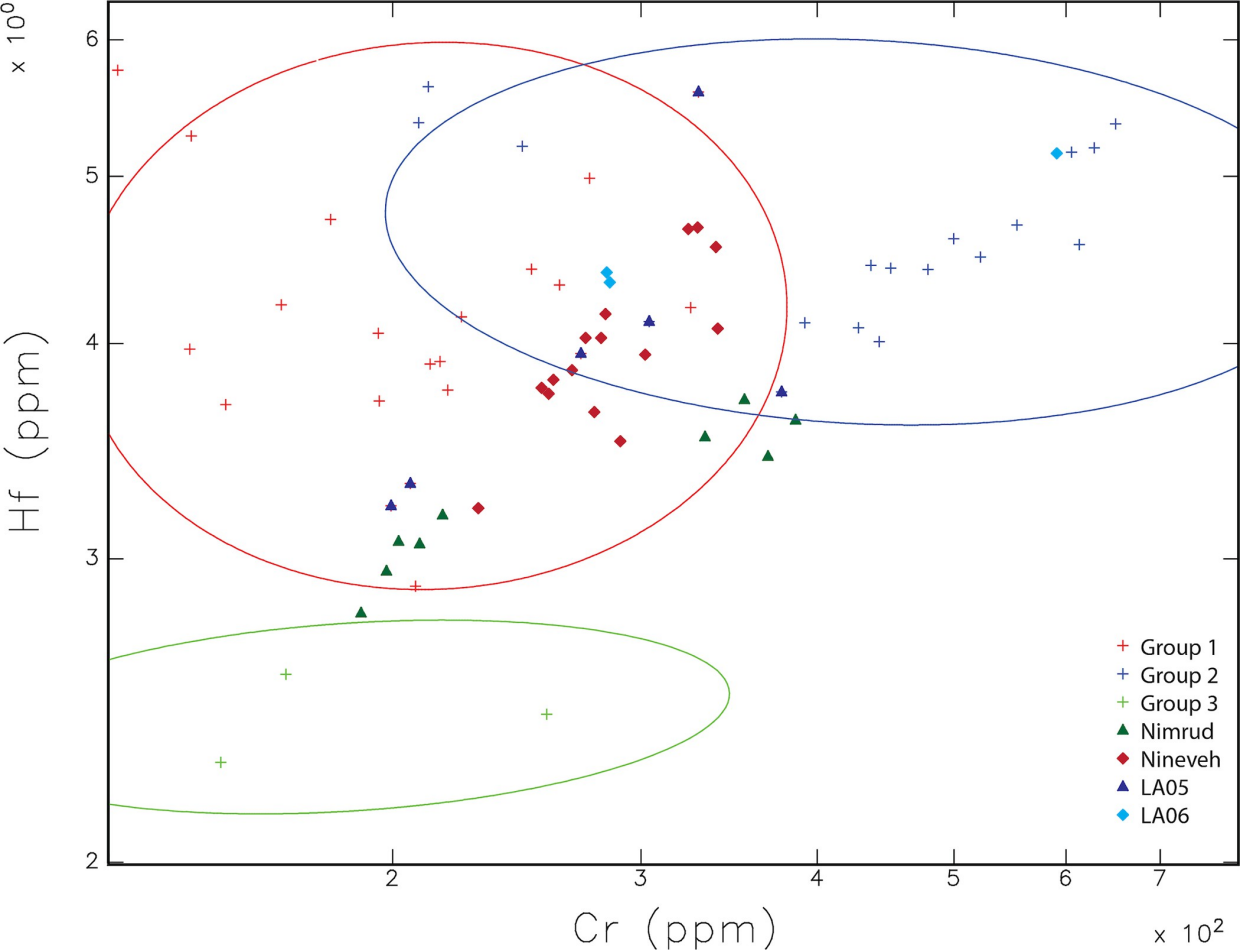
Scatterplot of Cr and Hf showing ellipses and samples from Ziyaret Tepe, along with samples from Nimrud and Nineveh. Ziyaret Tepe Palace Ware (LA05) samples are represented as dark blue triangles, and Near Palace Ware (LA06) samples are teal diamonds. Ellipses are drawn at 90% confidence intervals.

To detect further differences between the Ziyaret samples and those from the capitals and Dur-Katlimmu (Tell Sheikh Hamad), we noted that Hunt found that chromium (Cr) and hafnium (Hf) helped to differentiate some of her samples [2]. MURR then took these elements into consideration. Fig 8 below shows the same compositional ovals as above for Ziyaret but graphs the concentration of Hf versus Cr in each sample. It includes all Ziyaret samples (shown as plus signs) and highlights Ziyaret fine wares and fine wares from Nimrud and Nineveh as reported by Hunt [9]. The most obvious pattern is the overlap between the Ziyaret types and samples from the Assyrian heartland. Nimrud samples are green triangles and those from Nineveh are pink diamonds, and they either fall into Group 1 or 2, or just outside them with slightly less Hf. The Nimrud samples fall into two clusters, one with lower Cr and slightly lower Hf, and one with higher values of both. All of the samples from Nineveh fall completely within Group 1 or 2.

Two samples from Ziyaret (ZT 48352/1 and ZT 5738/3) plot closely to several samples from Nineveh and are also ones that were identified on other charts as near the Nineveh samples through PCA and Euclidean Distance analyses. Several other sherds of Palace or Near Palace Ware also plot closely to Nineveh in Fig 8. Their closeness was confirmed by MURR with a Euclidean distance search using the 25 elements analyzed in common for the samples between MURR and the Vienna lab. Overall, four Ziyaret samples (ZT 48352/1, ZT 503/8, ZT 503/2, and ZT 5738/3; see S1 Table) show the greatest similarity with the Nineveh samples from Hunt’s analysis. Since the Vienna lab did not measure Ca, a major component of the Ziyaret pottery, and the clays up and down the Tigris apparently exhibit very little variation in their major components, interpretation of these results are made with caution.

## Discussion and conclusion

In this study, we analyzed Plain Simple Ware and Palace Ware at a chemical level to characterize their similarities and differences. With the naked eye, it is easy to distinguish Palace Ware from the vast majority of other contemporary pottery, as it is clearly different in color, thickness, texture, and temper. However, such macroscopic distinguishing features do not necessarily mean the Palace Ware and more common pottery are made from different clay sources, or in far-flung workshops. Macroscopic variations could be due to the skill level of the potter, preparation processes for the clay, manufacturing (hand versus wheel), and firing techniques and conditions. Chemical studies of the clay body should show whether or not these two wares were made with different clays and tempers, thereby indicating multiple, geographically distinct clay sources and workshops.

One key contribution of our analysis is a chemical characterization of the local fabrics of pottery manufactured in the upper Tigris River valley in southeastern Turkey. Chemical studies have been conducted on clays in pottery from the Assyrian imperial capitals in northern Iraq and a few sites within 50 km of the imperial capitals along the Tigris. Other scholars have chemically analyzed clays at the western frontier of the Neo-Assyrian empire in the Levant. However, few have sampled the upper Tigris valley on the northern frontier of the empire, with the exception of Kibaroǧlu [23]. Statistical analyses of the chemical characterization places most of the Plain Simple Ware as well as the cooking pots into Groups 1 and 2, and additionally the presence of all the wasters in the same groups confirms that those groups represent the local recipe for pottery production used at Ziyaret.

An unforeseen result of the chemical analysis was the discovery of extra Ca in some of the Neo-Assyrian cooking pots. The MURR lab suggests that a production method involving slightly varied clay preparation would cause this chemical pattern of higher Ca, probably related to a need to create pots that could withstand thermal stress [35]. This result is promising and provides information on pottery production methods that is difficult to detect otherwise since we did not find Neo-Assyrian pottery workshops at Ziyaret.

Regarding finewares, it is likely that Palace Ware at Ziyaret Tepe was produced locally, imported, or both. There are few significant chemical differences between the clays of the upper Tigris river valley near Ziyaret and the Tigris river near the Assyrian capitals. It is therefore difficult to clearly separate imports from local products through NAA. If any are imported, Nineveh represents the most likely source among those discussed here, given the close proximity between its samples and several from Ziyaret in Fig 8.

Our initial hypothesis was that the finewares used at Neo-Assyrian Tušhan during the imperial period were made using local clay. The results of the NAA study undertaken to test this hypothesis do not provide any clear evidence to reject or revise this hypothesis. As noted earlier, if Palace Ware was made at Ziyaret, the larger significance would be that there are very highly skilled potters operating at a regional capital, as appears was the case at another regional capital, Tell Sheikh Hamad in Syria. A second confirmed case of highly skilled local potters indicates that the production of such an elite type of pottery was not restricted to workshops in the imperial heartland. Unlike other crafts such as metalworking and textile production, the Assyrian bureaucrats did not closely track the movements of finished ceramic vessels and, based on evidence presented here, appear to have allowed regional production either by highly skilled craftspeople brought in from the imperial heartland, or local imitators who followed the form and fashions set there, or both. Potters at Tušhan, Dur-Katlimmu and in other imperial peripheries therefore likely produced fineware pottery independent of direct government control.

In the future, sampling a greater variety of pottery from sites up and down the Tigris may make it possible to distinguish slight differences in the local compositions of clay used for pottery. Other researchers should take note of the proportion of rare earth elements such as chromium and hafnium, which may vary more significantly over the landscape than other more common elements. In our own ongoing research, we submitted NAA samples this year from two small farmstead sites in the Erbil Plain within the Assyrian heartland as part of the Sebittu Project. The samples were Plain Simple Ware as well as a few pieces of Palace Ware, to see how these vary chemically from the others already studied. Unlike a regional capital such as Tušhan or Dur-Katlimmu, we do not expect potters at such small sites to have produced fineware pottery themselves. We are likewise expanding our NAA study to include glazed wares from Tušhan in order to characterize this fineware chemically and see if it contrasts with Palace Ware. Broadly, glazed wares are even more rare than Palace Ware as their production required careful control of glaze preparation and precise temperature regulation as the vessels cooled after firing, in addition to other skilled manufacturing techniques. Further studies of the forms of ceramic vessels, glazed and otherwise, in comparison with vessels in metal or glass, may help us distinguish the relative value of each.

## Supporting information

S1 Table Raw NAA data. List of sample numbers by sample number and their elemental compositions, with additional tabs for PCA, total variation matrix calculation, and Mahalanobis distances.
(XLSX)

S1 File Inclusivity in global research. (DOCX)
